# Jucunde

**Published:** 1868-01-01

**Authors:** 


### JUCUNDE.

While we deem it of the first importance that the physician in the performance of his duties as a practitioner should consider, first, whether to give; next, what to give; then, how much to give, it is often of no less consequence that he consider well how to give his medicines. A bungling mixture may defeat the object of the most appropriate remedy. And it is worthy of commendation, that the attention of the profession, as well as pharmaceutists, is being directed especially to this too much neglected subject. The manufacture of granules and sugar-coated pills, containing not only the preparations of the pharmacopæia, but many of the more common prescriptions, is a stride in the right direction. Another, of no less value, is the preparation of the more usual and desirable combinations of bark, the various salts of iron, strychnia, and other drugs, in eligible forms for administration as elixirs, syrups, etc. These can often be better prepared in the laboratory of the chemist than on the table of the apothecary, and to be useful in practice, must contain the active principles of the drugs they represent, in precisely the quantities stated in the formulæ.

Many of this class of preparations found in the shops, from the employment of inferior drugs or other faults, are unreliable in strength, and practically worthless.

But while we favor the use of such preparations on the score of convenience and elegance, we can not refrain from a hearty condemnation of empiricism in any of its forms, whether on the part of a superficial or indolent practitioner, who glides into a mere routine, or the druggist who first offers his preparation to the profession, then recklessly gives it to the "public," basely quoting any favorable opinion he may have received from a medical gentleman to aid him in his illegitimate traffic.

We are led to make these remarks from noticing that the firm of Reed, Carnrick & Andrus, chemical manufacturers, New York, are now offering their products in this market. And from the reputation of the house, and the favor with which their medicinal compounds have been received by the profession, where they have been used and thoroughly tested, we believe they are well deserving confidence, and will prove of value to the careful practitioner.

